# Non-linear associations of serum spermidine with type 2 diabetes mellitus and fasting plasma glucose: a cross-sectional study

**DOI:** 10.3389/fnut.2024.1393552

**Published:** 2024-05-15

**Authors:** Xiaohong Zhang, Yao Zhang, Shaojie Li, Min Liu, Ying Lu, Mengyao He, Zhaoqing Sun, Mingfeng Ma, Liqiang Zheng

**Affiliations:** ^1^Clinical Research Centre, The International Peace Maternity and Child Health Hospital, Shanghai Jiao Tong University School of Medicine, Shanghai, China; ^2^School of Public Health, Shanghai Jiao Tong University School of Medicine, Shanghai, China; ^3^Ministry of Education-Shanghai Key Laboratory of Children’s Environmental Health, Xinhua Hospital Affiliated to Shanghai Jiao Tong University School of Medicine, Shanghai, China; ^4^Department of Endocrinology, Shengjing Hospital of China Medical University, Shenyang, China; ^5^Department of Cardiovascular Medicine, Fenyang College of Shanxi Medical University, Fenyang Hospital Affiliated to Shanxi Medical University, Fenyang, Shanxi, China; ^6^Department of Biostatistics and Epidemiology, School of Public Health, China Medical University, Shenyang, China; ^7^Department of Physical and Chemical, Shanghai Changning District Center for Disease Control and Prevention, Shanghai, China; ^8^Department of Cardiology, Shengjing Hospital of China Medical University, Shenyang, China; ^9^Department of Cardiovascular Medicine, Fenyang Hospital Affiliated to Shanxi Medical University, Fenyang, Shanxi, China; ^10^Hainan Branch, Shanghai Children’s Medical Center, School of Medicine, Shanghai Jiao Tong University, Sanya, China

**Keywords:** spermidine, T2DM, FPG, non-linear association, cross-sectional study

## Abstract

**Background:**

Previous animal experiments have demonstrated the potential of spermidine to mitigate glucose intolerance, insulin resistance, and hyperinsulinemia. However, there remains a scarcity of epidemiological evidence supporting these findings. Therefore, we aimed to elucidate the associations of serum spermidine with T2DM and FPG.

**Materials and methods:**

The cross-sectional study was conducted from June to August 2019 in the rural areas of Fuxin County, Liaoning Province, China. A total of 4,437 participants were included in the study. The serum spermidine was detected using high-performance liquid chromatography with a fluorescence detector. FPG was measured using the hexokinase method. T2DM was defined as participants with a FPG level of 7.0 mmol/L or greater, or self-reported diagnosis of diabetes by a doctor. Restricted cubic spline model and piecewise linear regression model were used to explore the associations of serum spermidine with T2DM and FPG, respectively.

**Results:**

The mean (SD) age of the participants was 59.3 (10.0) years, with 622 out of 4,437 participants being defined as T2DM. The serum spermidine in participants stratified by age and BMI categories was significantly different, with *p* values of 0.006 and 0.001, respectively. Among all the participants, the association of serum spermidine with T2DM was J-shaped. The log (spermidine) was negatively associated with T2DM (OR = 0.68, 95% CI: 0.52 to 0.92, *p* = 0.01) below the inflection point, while log (spermidine) was not significantly associated with T2DM (OR = 1.97, 95% CI: 0.93 to 4.15, *p* = 0.07) above the inflection point. Among the participants without T2DM, the association of serum spermidine with FPG was inverted J-shaped. The log (spermidine) was positively associated with FPG (*β* = 0.13, 95% CI: 0.05 to 0.21, *p* = 0.001) below the inflection point, while log (spermidine) was negatively associated with FPG (*β* = −0.29, 95% CI: −0.42 to −0.16, *p* < 0.001) above the inflection point.

**Conclusion:**

In conclusion, non-linear associations of serum spermidine with T2DM and FPG were found in the cross-sectional study in Chinese rural adults. This provided insights into the use of spermidine for the prevention of T2DM, highlighting the potential role in public health prevention strategies of spermidine.

## 1 Introduction

The global prevalence of type 2 diabetes mellitus (T2DM) and its associated complications represents a significant public health concern. According to the International Diabetes Federation (IDF), an estimated 10.5% of adults aged 20–79 years, equating to 537 million individuals, were living with diabetes mellitus in 2021 (1). Projections indicate that this figure is expected to increase to 783 million by 2045, with a 16% rise in prevalence attributed to the aging of the population (1).

Spermidine, a natural polyamine, is derived from oral uptake with the food, production by intestinal microorganisms and cellular biosynthesis (2). Its presence varies among different species, cell types, and tissues, as well as among foods of animal, fungi, or plant origin (3). Studies in model organisms and humans have shown that tissue spermidine concentrations have been observed to decrease with age (2, 4, 5). Furthermore, it contains three positively charged amino groups, and this structural characteristic is crucial for its functions, which include stabilizing DNA, promoting cell growth, and inducing autophagy (6). Being a natural autophagy inducer, spermidine has displayed pleiotropic health effects, such as lifespan extension (7), tumor inhibition (8), cardiovascular protection (9), neuromodulation (10) and weight loss (11).

Previous studies in rodent animal models presented spermidine can modulate glucose homeostasis (12). Mutant mice with elevated spermidine levels exhibited reduced circulating glucose levels compared to controls, suggesting protection from diet-induced T2DM by spermidine (13). Additionally, the increase of glycosylated hemoglobin A1c (HbA1C) in diabetic rats was arrested with spermidine treatment (14). In additional, daily administration of spermidine to mice eating a high-fat diet ameliorated glucose intolerance, insulin resistance, and hyperinsulinemia (15). Similar to the findings in animals, spermidine calculated from dietary intake in a general population of National Health and Nutrition Examination Survey (NHANES) from 2009 to 2010 was negatively associated with serum glucose, insulin, HbA1C levels, and homeostatic model assessment of insulin resistance (HOMA-IR) index (15). Furthermore, our population-based study showed serum spermidine was inversely associated with triglyceride-glucose index and the risk of insulin resistance (16). However, to our knowledge, the associations between spermidine and T2DM and fasting plasma glucose (FPG) has not been fully explored. Therefore, our study aimed to examine the associations of serum spermidine with T2DM and FPG using a Chinese rural population-based cohort.

## 2 Materials and methods

### 2.1 Study design and participants

This study utilized data from a large-scale cohort study that was originally designed as a cardiometabolic cohort to investigate the association between single and clustered cardiovascular risk factors and mortality. The non-randomized cross-sectional survey was conducted in the rural areas of Fuxin County, Liaoning Province, China, from June to August 2019. According to the geographical distribution of Fuxin County, the area was divided into three regions: eastern, southern, and northern. Based on the population size and transportation convenience among different townships, two townships were selected from the southern region, while one township was chosen from both the northern and eastern regions using a computer-generated simple random sampling procedure. Then eligible villages and villagers who met the inclusion criteria were invited to participate in this study. Ultimately, a total of 33 villages and 4,689 villagers were selected for the cross-sectional survey. The survey included a questionnaire survey, physical examinations (including height, weight, waist circumference, hip circumference, blood pressure and so on), and biochemical blood tests.

The study’s inclusion criteria for participants were as follows: (1) aged 35 years or older (17, 18); (2) no pregnancy; (3) no severe liver and renal failure; (4) having resided locally for at least 5 years; and (5) having signed informed consent. The exclusion criteria included: (1) missing the test of serum spermidine; (2) missing the message of age, sex, educational level, income, BMI and FPG; and (3) unwillingness to participate in this study. All participants provided written informed consent prior to study inclusion. If the participants were unable to write, their guardians would read and sign the informed consent on behalf of them.

### 2.2 Measurement of serum spermidine and FPG

Participants were required to fast for at least 8 h prior to undergoing the examinations and providing blood samples. Fasting blood samples were obtained in the morning by drawing from the antecubital vein and collected in siliconized vacuum glass tubes. The collection of fasting blood samples was performed at a centralized location in each village. After blood samples were processed, they were sent to Shenyang Di’an Medical Laboratory Co., Ltd. for biochemical analysis. FPG was measured using a Roche Cobas 8,000 C701 automated biochemistry analyzer with the hexokinase method. Serum was obtained by centrifugation at 3,000 rpm for 10 min and then stored at −80°C until further analysis. The measurement of serum spermidine was conducted in laboratory platform of Quality and Safety Testing Center, Institute of Agro-Products Processing, Chinese Academy of Agricultural Sciences. The detailed process of detecting serum spermidine was described in the previous study (12). Briefly, blood samples were drawn from the antecubital vein in the morning and were collected into siliconized vacuum glass tubes (Corning, NY, USA). Serum was obtained by centrifugation at 3,000 rpm for 10 min and then stored at −80°C until further analysis. Serum spermidine was measured using high-performance liquid chromatography (Thermo fisher scientific, MA, USA).

### 2.3 Definition of T2DM

T2DM was defined as participants with a FPG level of 7.0 mmol/L or greater, or self-reported diagnosis of diabetes by a doctor from a township health center, community service center, or higher-level medical institution. The diagnosis of diabetes was self-reported by participants in face-to-face interviews using the question “Have you ever been diagnosed with diabetes by a doctor from a township health center, community service center, or higher-level medical institution? Note for the interviewer: Excluding gestational diabetes. The diagnosis should be accompanied by diagnosis certificate of a medical institution.” If the answer was yes, self-reported diagnosis of diabetes by a doctor was yes. If the answer was no, self-reported diagnosis of diabetes by a doctor was no.

### 2.4 Assessment and definition of other variables

Data on sociodemographic and lifestyle characteristics were collected using standardized questionnaires in face-to-face interviews, including data on age, sex (male, female), and ethnicity (Han ethnicity, Minorities ethnicity), educational level (never, primary school, junior high school, senior high school or higher education), income ($ < 1,450, $1,450 ~ 4,349, $ ≥ 4,349). The face-to-face interview was conducted at a centralized location in each village. During face-to-face interviews, participants were asked about their income in categories of <10,000 RMB, 10,000–30,000 RMB, and ≥ 30,000 RMB. To facilitate comparison with international literature, the income categories were converted into $ < 1,450, $1,450-4,349, and $ ≥ 4,349 according to the average exchange rate between RMB and USD in 2019, 6.8985 RMB per USD. Height and weight were measured by trained staff using body measurement instruments (Sidi RGZ-120, Changzhou, China) at a centralized location in each village, and the results were promptly recorded in the corresponding sections of the questionnaire. The instrument was carefully inspected and strictly calibrated before each use to ensure its accuracy and precision. During the measurement, participants should remove shoes, hats, outerwear, and wear thin clothing. The measurement value was read from the digital display. Height was measured with an accuracy of 0.1 centimeter and weight was measured with an accuracy of 0.1 kilograms. Body mass index (BMI) was calculated as weight in kilograms divided by height in meters squared. If BMI < 18.5 kg/m^2^, BMI group was defined as underweight. If 18.5 ≤ BMI < 24 kg/m^2^, BMI group was defined as normal. If 24 ≤ BMI < 28 kg/m^2^, BMI group was defined as overweight. IF BMI ≥ 28 kg/m^2^, BMI group was defined as obese.

### 2.5 Statistical analysis

Participants were separated into two groups based on whether they had T2DM. Categorical variables were expressed as counts and percentages and the differences were tested by Chi-Square tests. Normal-distributed continuous variables were described by means (SD) and the differences were tested by Student t test. Skewed-distributed continuous variables were described by median (Q1-Q3) and the differences were tested by non-parametric analysis. Baseline variables differences were tested by Student t test and Chi-Square tests.

Logistic regression models, which incorporated with restricted cubic splines with 4 knots (20th, 40th, 60th, and 80th percentiles) of serum spermidine and two-piecewise linear regression models with log (spermidine), were used to explore the nonlinear associations of serum spermidine with T2DM (a binary variable) among all the participants. For FPG, a continuous quantitative variable, linear regression models were used to explore the nonlinear associations, which incorporated with restricted cubic splines with 4 knots (20th, 40th, 60th, and 80th percentiles) of serum spermidine and two-piecewise linear regression models with log (spermidine). As serum spermidine did not follow a normal distribution, it was log-transformed to achieve normality and used in two-piecewise linear regression models. Model 1 was adjusted for sociodemographic factors, including age, sex (male, female), educational level (never, primary school, junior high school, senior high school or higher education) and income ($ < 1,450, $1,450 ~ 4,349, $ ≥ 4,349); Model 2 added to model 1 the body mass index (BMI) group (underweight, normal, overweight, obese) as covariates. The reference category for each categorical variable included in the adjusted models was the first level, which was first described in Table 1.

**Table 1:** Baseline characteristics of study population by presence of T2DM.^*^

Characteristics	Total	Without T2DM	With T2DM	*p* value
No. (%)	4,437	3,815 (86.0)	622 (14.0)	
Spermidine, median (Q1–Q3), ng/ml	24.8 (13.4–50.4)	24.8 (13.6–50.3)	24.8 (12.5–50.5)	0.36
Age, year	59.3 ± 10.0	59.0 ± 10.2	61.1 ± 8.2	<0.001
Age group, no. (%)				<0.001
<60	2,137 (48.2)	1894 (88.6)	243 (11.4)	
≥60	2,300 (51.8)	1921 (83.5)	379 (16.5)	
Sex, no. (%)				<0.001
Male	1,595 (35.9)	1,413 (88.6)	182 (11.4)	
Female	2,842 (64.1)	2,402 (84.5)	440 (15.5)	
Ethnicity, no. (%)				0.72
Han ethnicity	2,908 (65.6)	2,505 (86.1)	403 (13.9)	
Minorities ethnicity	1,523 (34.4)	1,306 (85.8)	217 (14.2)	
Educational level, no. (%)				0.004
Never	181 (4.1)	148 (81.8)	33 (18.2)	
Primary school	1776 (40.0)	1,495 (84.2)	281 (15.8)	
Junior high school	1875 (42.3)	1,645 (87.7)	230 (12.3)	
Senior high school or higher education	605 (13.6)	527 (87.1)	78 (12.9)	
Income, dollars, no. (%)				<0.001
$ < 1,450	3,023 (70.5)	2,562 (84.8)	461 (15.2)	
$1,450 ~ 4,349	1,034 (24.1)	923 (89.3)	111 (10.7)	
$ ≥ 4,349	231 (5.4)	202 (87.4)	29 (12.6)	
BMI group^†^, no. (%)				<0.001
Underweight	165 (3.7)	156 (94.5)	9 (5.5)	
Normal	1748 (39.4)	1,547 (88.5)	201 (11.5)	
Overweight	1719 (38.7)	1,443 (84.0)	276 (16.0)	
Obese	805 (18.1)	669 (83.1)	136 (16.9)	
Fasting plasma glucose, mmol/L	6.0 ± 1.9	5.4 ± 0.6	9.3 ± 3.1	<0.001

Q1, the first quartile; Q3, the third quartile; BMI, body mass index.

^*^Unless otherwise indicated, data are expressed as mean ± SD.

^†^BMI was calculated as weight in kilograms divided by height in meters squared. Underweight: BMI < 18.5 kg/m^2^; Normal: 18.5 ≤ BMI < 24 kg/m^2^; Overweight: 24 ≤ BMI < 28 kg/m^2^; Obese: BMI ≥ 28 kg/m^2^.

In sensitivity analyses, missing variables were handled by imputing the modal number of the variables. Specifically, missing data for educational level, income, and BMI group were imputed with the modal values (“junior high school” for educational level, “<1,450 dollars” for income, and “normal” for BMI group). However, missing data for age and sex of 56 individuals were not imputed as they only had values for spermidine and lacked information on other variables. Additionally, missing data FPG of one individual was not imputed yet. Fully adjusted logistic regression models and linear regression models, incorporating restricted cubic splines with 4 knots (20th, 40th, 60th, and 80th percentiles), were employed to analyze the association of serum spermidine levels with the presence of T2DM in participants, as well as the association with FPG in participants without T2DM.

Statistical analyses were performed using SAS version 9.4 (SAS Institute Inc., Cary, NC, USA), while restricted cubic splines model and two-line piecewise linear model were performed using R (version 4.2.2).^1^ A two-tailed *p*-value of less than 0.05 was considered statistically significant.

## 3 Results

### 3.1 Study participants

**Figure 1:**
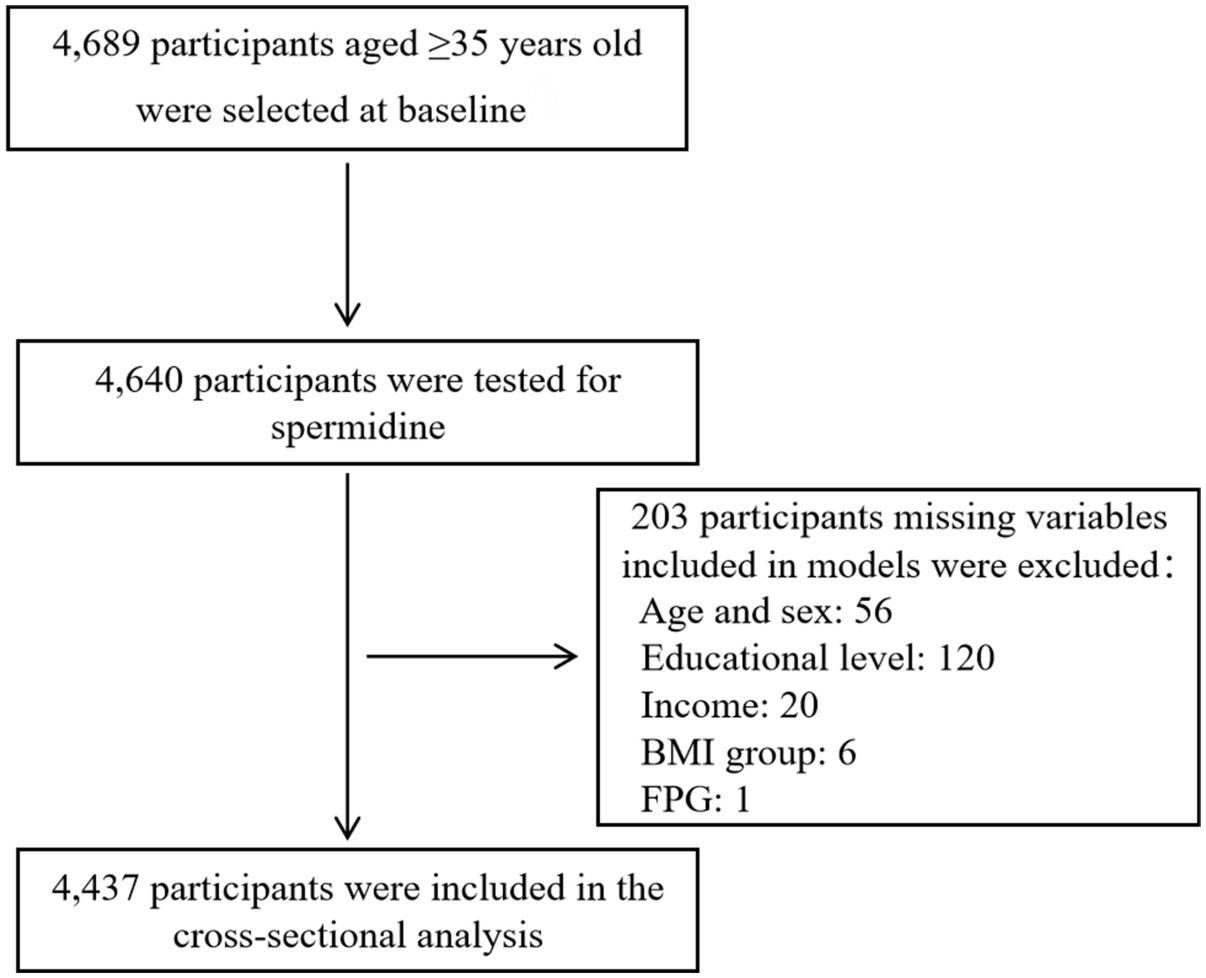
Flow chart of participants included in the cross-sectional analysis.

A total of 4,689 individuals were included in this study, of which 4,640 individuals (99.0%) underwent testing for serum spermidine. 203 individuals (4.4%, age and sex: 56, educational level: 120, income: 20, BMI group: 6, FPG: 1) were excluded due to missing variables, resulting in leaving a final analysis of 4,437 participants. The flow-chart was showed in Figure 1. Sociodemographic and clinical information was summarized in Table 1. The mean [SD] age of the participants was 59.3 [10.0] years, with 64.1% being women, 65.6% belonging to the Han ethnicity, and 622 diagnosed with T2DM. The median (Q1–Q3) of serum spermidine in non-T2DM group and T2DM group was 24.8 (13.6–50.3) ng/ml and 24.8 (12.5–50.5) ng/ml, respectively, with a *p*-value of 0.36. Apart from ethnicity, we found significant differences in age, sex, educational level and BMI group between participants without T2DM and those with T2DM.

Additionally, to better analyze the levels of serum spermidine in the study population, the levels of serum spermidine in participants stratified by age and BMI categories, both in those without and with T2DM, are presented in Table 2. The levels of serum spermidine among participants under 60 years old and those over 60 years old were 24.3 (13.2–46.6) ng/ml and 26.8 (13.6–52.7) ng/ml, respectively. The difference of serum spermidine between age groups was found to be statistically significant, with a *p*-value of 0.006. Similarly, the levels of serum spermidine among BMI groups were 20.9 (11.8–36.5) ng/ml for underweight, 24.3 (12.8–47.8) ng/ml for normal weight, 25.3 (13.8–50.5) ng/ml for overweight and 27.7 (14.9–54.5) ng/ml for obese participants. The difference of serum spermidine between BMI group was also significant, with a *p* value of 0.001. Among participants with normal weight, the levels of serum spermidine were 24.8 (13.1–48.6) ng/ml in those without T2DM and 18.9 (11.4–36.7) ng/ml in those with T2DM. The difference of serum spermidine was significant, with a p value of 0.002. Apart from these findings, the differences in serum spermidine levels among participants stratified by age and BMI categories, as well as by the presence of T2DM, were not found to be statistically significant.

**Table 2:** The levels of serum spermidine in the participants stratified by age and BMI categories and by presence of T2DM.^*^

	Total	Without T2DM	With T2DM	*p* value
Age group				0.006^‡^
<60	24.3 (13.2–46.6)	24.7 (13.4–47.0)	23.1 (11.7–44.6)	0.15^§^
≥60	26.8 (13.6–52.7)	26.8 (13.8–52.5)	26.8 (13.0–56.4)	0.80^§^
BMI group^†^				0.001^‡^
Underweight	20.9 (11.8–36.5)	20.9 (12.0–36.7)	20.3 (11.8–34.5)	0.79^§^
Normal	24.3 (12.8–47.8)	24.8 (13.1–48.6)	18.9 (11.4–36.7)	0.002^§^
Overweight	25.3 (13.8–50.5)	24.8 (13.9–50.5)	26.8 (13.6–52.5)	0.95^§^
Obese	27.7 (14.9–54.5)	27.2 (14.1–52.9)	30.8 (15.6–60.7)	0.95^§^

Q1, the first quartile; Q3, the third quartile; BMI, body mass index.

^*^The levels of spermidine were expressed as median (Q1-Q3) ng/ml.

^†^BMI was calculated as weight in kilograms divided by height in meters squared. Underweight: BMI < 18.5 kg/m^2^; Normal: 18.5 ≤ BMI < 24 kg/m^2^; Overweight: 24 ≤ BMI < 28 kg/m^2^; Obese: BMI ≥ 28 kg/m^2^.

^‡^The *p*-values indicate the statistical significance of differences in serum spermidine levels between age groups and BMI groups, as determined by non-parametric analysis.

^§^The *p*-values indicate the statistical significance of differences in serum spermidine levels between individuals without T2DM and those with T2DM across different subgroups of age and BMI, as determined by non-parametric analysis.

### 3.2 Non-linear associations of serum spermidine with T2DM among all the participants and FPG among the participants without T2DM by restricted cubic splines model

**Figure 2:**
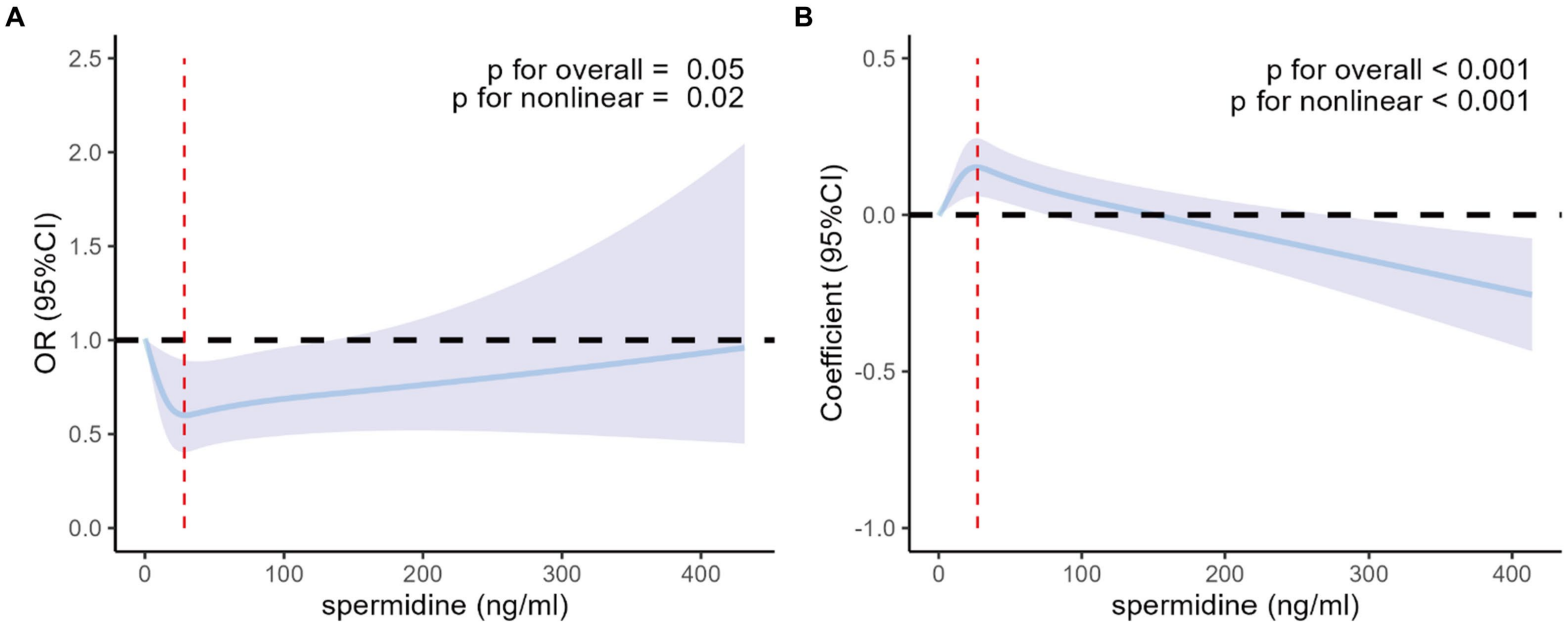
Nonlinear association of serum spermidine with T2DM among all the participants and with FPG among participants without T2DM. **(A)** Nonlinear association between serum spermidine and T2DM among all the participants. Odds ratio was indicated by solid lines and 95% CIs by shaded areas. The red dashed line indicates the value of serum spermidine at the inflection point (spermidine = 28.4 ng/mL). **(B)** Nonlinear association between serum spermidine and FPG among participants without T2DM. Coefficient was indicated by solid lines and 95% CIs by shaded areas. The red dashed line indicates the value of serum spermidine at the inflection point (spermidine = 27.2 ng/mL). The models were adjusted for age, sex, education level, income, BMI group. T2DM, type 2 diabetes mellitus; FPG, fasting plasma glucose; OR, odds ratio; CI, confidence interval; BMI, body mass index.

In the fully adjusted logistic regression model incorporating restricted cubic splines with 4 knots (20th, 40th, 60th, and 80th percentiles) of serum spermidine, the nonlinear association of serum spermidine with T2DM among all the participants was found to be J-shaped, as depicted in Figure 2A. The *p* values for overall and non-linearity of the J-shaped association were 0.05 and 0.02, respectively. In the fully adjusted linear regression model incorporating restricted cubic splines with 4 knots (20th, 40th, 60th, and 80th percentiles) of serum spermidine, the nonlinear association of serum spermidine with FPG among the participants without T2DM was found to be inverted J-shaped, as depicted in Figure 2B. The *p* values for overall and non-linearity of the inverted J-shaped association were both <0.001. The concentration of serum spermidine at the inflection points in the J-shaped association and in the inverted J-shaped association were 28.4 ng/mL and 27.2 ng/mL, respectively.

### 3.3 Associations of serum spermidine with T2DM among all the participants and FPG among the participants without T2DM by two-line piecewise linear model

In multivariable logistic regression models that adjusted by different confounding variables incorporating with two-line piecewise linear model, the associations between log (spermidine) and T2DM among all the participants were negative below the inflection point (model 1: OR = 0.69, 95% CI: 0.52 to 0.92, *p* = 0.01; model 2: OR = 0.68, 95% CI: 0.52 to 0.92, *p* = 0.01), while the association were positive above the inflection point in model 1 (OR = 2.19, 95% CI: 1.04 to 4.60, *p* = 0.001) and had no significance in model 2 (OR = 1.97, 95% CI: 0.93 to 4.15, *p* = 0.07), with the inflection point of 1.71 of log (spermidine), as depicted in Table 3. Furthermore, in multivariable linear regression models that adjusted by different confounding variables incorporating with two-line piecewise linear model, the associations between log (spermidine) and FPG among the participants without T2DM were positive below the inflection point (model 1: *β* = 0.13, 95% CI: 0.05 to 0.21, *p* = 0.002; model 2: *β* = 0.13, 95% CI: 0.05 to 0.21, *p* = 0.001), while the associations were negative above the inflection point (model 1: *β* = −0.26, 95% CI: −0.40 to −0.12, *p* < 0.001; model 2: *β* = −0.29, 95% CI: −0.42 to −0.16, *p* < 0.001), with the inflection point of 1.56 of log (spermidine), as depicted in Table 4.

**Table 3:** Nonlinear association between serum spermidine and T2DM among all the participants.

Models	The inflection points of log (spermidine)	Below the inflection point		Above the inflection point	
		ORs (95% CI)	*p* value	ORs (95% CI)	*p* value
Model 1^*^	1.71	0.69 (0.52 to 0.92)	0.01	2.19 (1.04 to 4.60)	0.001
Model 2^†^	1.71	0.68 (0.52 to 0.92)	0.01	1.97 (0.93 to 4.15)	0.07

OR, odds ratio; CI, confidence interval.

^*^Model 1 was adjusted for age, sex, educational level, income.

^†^Model 2 was further adjusted for BMI group.

**Table 4:** Nonlinear association between serum spermidine and FPG among the participants without T2DM.

Models	The inflection points of log (spermidine)	Below the inflection point		Above the inflection point	
		Coefficient (*β*) (95% CI)	*p* value	Coefficient (*β*) (95% CI)	*p* value
Model 1^*^	1.56	0.13 (0.05 to 0.21)	0.002	−0.26 (−0.40 to −0.12)	<0.001
Model 2^†^	1.56	0.13 (0.05 to 0.21)	0.001	−0.29 (−0.42 to −0.16)	<0.001

CI, confidence interval.

^*^Model 1 was adjusted for age, sex, educational level, income.

^†^Model 2 was further adjusted for BMI group.

### 3.4 Sensitive analysis

**Figure 3:**
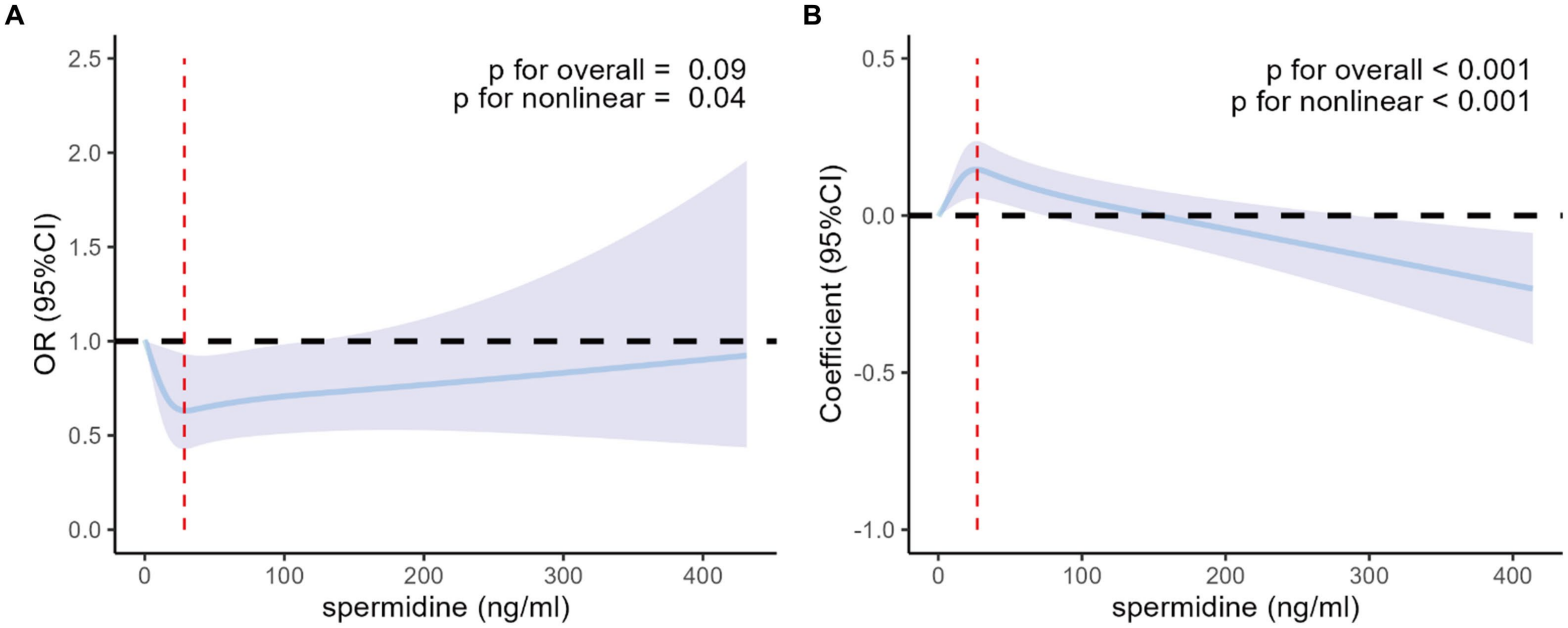
Nonlinear association of serum spermidine with T2DM among the participants included in the sensitive analyses and with FPG among those without T2DM. **(A)** Nonlinear association between serum spermidine and T2DM among the participants included in the sensitive analyses. Odds ratio was indicated by solid lines and 95% CIs by shaded areas. The red dashed line indicates the value of serum spermidine at the inflection point (spermidine = 28.4 ng/mL). **(B)** Nonlinear association between serum spermidine and FPG among participants without T2DM included in the sensitive analyses. Coefficient was indicated by solid lines and 95% CIs by shaded areas. The red dashed line indicates the value of serum spermidine at the inflection point (spermidine = 27.2 ng/mL). The models were adjusted for age, sex, education level, income, BMI group. T2DM, type 2 diabetes mellitus; FPG, fasting plasma glucose; OR, odds ratio; CI, confidence interval; BMI, body mass index.

4,583 individuals were included in sensitive analyses. In the fully adjusted models incorporating restricted cubic splines with 4 knots (20th, 40th, 60th, and 80th percentiles) of serum spermidine, as depicted in Figure 3, the nonlinear associations of serum spermidine with T2DM among the analyzed participants and with FPG among those without T2DM remained consistent with the primary findings of this study.

## 4 Discussion

In this cross-sectional study, we found non-linear associations of serum spermidine with T2DM among all the participants and with FPG among the participants without T2DM. Additionally, the association of log (spermidine) with T2DM among all the participants was negative below the inflection point, and had no significance in fully-adjusted model above the inflection point. The log (spermidine) was positively associated with FPG among the participants without T2DM below the inflection point, and was negatively above the inflection point. To the best of our knowledge, this is the first study to examine the associations of serum spermidine with T2DM and FPG.

Though the epidemiological study on the association between spermidine and T2DM was limited, there have been other epidemiological studies on the associations between spermidine and various diseases (9, 15, 19–21). In Bruneck Study, a prospective population-based study, higher intake of dietary spermidine as assessed from food-frequency questionnaires was found to be correlated with reduced blood pressure, a lower incidence of cardiovascular disease (9), enhanced cognition (20) and lower mortality (19). Moreover, increased dietary spermidine was associated with a decreased risk of cardiovascular disease and all-cause mortality in the U.S. population recruited in the NHANES from 2003 to 2014 (21). In addition to the obesity-associated parameters mentioned before, daily intake of spermidine was negatively correlated with body mass index and waist circumference in the NHANES from 2009 to 2010 (15). However, it’s important to note that self-reported dietary information obtained from food frequency questionnaires is susceptible to measurement error, which could be attributed to recall bias and social desirability. Therefore, dietary spermidine intake may not entirely reflect the actual spermidine concentration in the body. The present study aims to examine serum spermidine levels as an objective reflection of the concentration of spermidine in the body, providing a more reliable basis for subsequent promotion and application. Additionally, in the J-shaped association between serum spermidine and the risk of T2DM, it was observed that the risk of T2DM increased with serum spermidine levels increasing above the inflection point. We hypothesized that the trend was a compensatory effect in response to a pathological state. In other words, during the development of T2DM, the body may increase the synthesis or intake of spermidine to compensate for the loss of certain functions or organs. However, when spermidine levels become excessively high, it may have a negative impact and lead to an increased risk of T2DM. Similarly, the differences in serum spermidine levels among age groups can also be attributed to pathological state.

Spermidine, as a type of caloric restriction mimetic and natural autophagy inducer, has been demonstrated to exert these effects in various model organisms (2, 7, 22). Studies have shown that genetic ablation of autophagy abolishes spermidine-mediated lifespan extension in yeast, nematodes, and flies, and diminishes the cardioprotective effects in mice (9). The inhibitory effect of spermidine on several acetyltransferases, including EP300, has been linked to autophagy induction (23, 24). Moreover, spermidine has been found to inhibit mTORC1 and activate AMPK (25). It has been suggested that spermidine may post-translationally hypusinate the translation factor eIF5A, leading to the synthesis of the pro-autophagy transcription factor TFEB, particularly in immune cells (26). Moreover, spermidine has been shown to promote mitophagy, a specialized form of autophagy responsible for eliminating damaged or dysfunctional mitochondria, in both cell culture and mice (9, 27). A recent study has demonstrated that spermidine can alter the composition and function of the gut microbiota to ameliorate high-fat diet-induced metabolic syndrome by inducing autophagy (15). Although the exact mechanisms through which spermidine might influence type 2 diabetes mellitus are not fully understood, the potential mechanisms underlying the association between serum spermidine and type 2 diabetes mellitus may be partially explained by autophagy. It is important to note that while these findings are compelling, studies on the association between spermidine and T2DM remain limited, highlighting the need for further research in this area.

In contrast to preceding investigations, this study exhibits two notable strengths. Primarily, rather than solely evaluating dietary spermidine intake, the examination encompasses a comprehensive assessment of overall spermidine levels within the body, thereby accounting for all potential sources of spermidine. Simultaneously, the quantification of spermidine in serum mitigates the impact of recall bias inherent in dietary assessment scales. Secondly, in contrast to the dietary spermidine data obtained from 1908 participants in the NHANES study conducted from 2009 to 2010 (15), this study pioneers an exploration into the associations between serum spermidine levels and both T2DM and FPG within a substantially larger sample size comprising 4,437 individuals from the general population.

However, several noteworthy limitations warrant consideration in this study. Primarily, the cross-sectional nature of the research precludes establishing a causal relationship between serum spermidine levels and T2DM. Additionally, the single-time collection of blood samples may not adequately represent long-term status, potentially leading to measurement errors. The absence of meticulous control over diabetes diagnosis and its severity within the analysis is notable due to insufficient data regarding key factors such as diabetes duration, specific medications, and HbA1c levels. Furthermore, the absence of an assessment of dietary spermidine intake poses a challenge in correlating *in vivo* spermidine levels with serum spermidine levels. This absence restricts our ability to establish a direct correspondence between these variables. Moreover, the exclusive examination of the Chinese rural population might limit the generalizability of the findings. Considering the significant variability in serum spermidine levels across diverse populations due to differing sources of spermidine, a more comprehensive investigation involving diverse demographic groups would be pivotal to validate and extend these findings. Equally important, the study was conducted with adults ≥35 years old and it is therefore not possible to extrapolate the results to other age categories.

In conclusion, this study represents the inaugural report, to the best of our knowledge, of the non-linear association of serum spermidine levels with T2DM and FPG in a non-randomized cross-sectional analysis of Chinese rural adults. This provided insights into the use of spermidine for the prevention of T2DM, highlighting the potential role in public health prevention strategies of spermidine. Nevertheless, it is imperative to underscore the necessity for further investigations to establish the causal relationship between spermidine and T2DM.

## Data availability statement

The raw data supporting the conclusions of this article will be made available by the authors, without undue reservation.

## Ethics statement

The studies involving humans were approved by Shanghai Jiao Tong University School of Medicine. The studies were conducted in accordance with the local legislation and institutional requirements. The human samples used in this study were acquired from primarily isolated as part of your previous study for which ethical approval was obtained. Written informed consent for participation was not required from the participants or the participants’ legal guardians/next of kin in accordance with the national legislation and institutional requirements.

## Author contributions

XZ: Formal analysis, Writing – original draft, Writing – review & editing. YZ: Investigation, Writing – review & editing. SL: Investigation, Writing – review & editing. ML: Project administration, Writing – review & editing. YL: Project administration, Writing – review & editing. MH: Project administration, Writing – review & editing. ZS: Writing – review & editing. MM: Funding acquisition, Writing – review & editing. LZ: Data curation, Funding acquisition, Writing – review & editing.

## Footnotes

^1^ http://www.R-project.org
